# Primary vs. revision total elbow arthroplasty: an updated analysis of short-term complications and associated factors

**DOI:** 10.1016/j.jsea.2026.100036

**Published:** 2026-05-12

**Authors:** James R. Satalich, Matthew S. Smith, Sashrik Sribhashyam, Jenna L. Hughes, Mohini Johri, Benjamin P. Cassidy, Jennifer L. Vanderbeck, Shahabeddin Yazdanpanah

**Affiliations:** a Department of Orthopaedic Surgery, Virginia Commonwealth University, Richmond, VA, USA; b College of Medicine, Northeast Ohio Medical University, Rootstown, OH, USA

**Keywords:** Total elbow arthroplasty, Total elbow replacement, Revision, Complication, Adverse event, Infection, Operative time, NSQIP

## Abstract

**Background:**

Total elbow arthroplasty (TEA) is an increasingly utilized intervention for a range of pathologies. While the current literature landscape has emphasized reimbursement and long-term outcomes, for example, contemporary comparative data on short-term adverse events between primary and revision TEA remain limited. Therefore, this study updates and expands upon such short-term findings using a large database.

**Methods:**

In this retrospective cohort study, patients undergoing primary or revision TEA were identified in the American College of Surgeons National Surgical Quality Improvement Program database (2014-2023). Cases with missing data or complex additional/concurrent procedures were excluded. The primary outcome was a composite of any adverse event (AAE), alongside other subgroupings. Statistics included chi-square or Fisher's exact tests, independent *t*-tests, exploratory multivariable logistic regressions with odds ratios (ORs), and threshold analyses.

**Results:**

A total of 583 patients underwent primary TEA (mean age = 66.4 ± 13.3; body mass index = 29.9 ± 7.2 kg/m^2^; 21.3% male) and 179 underwent revision TEA (age = 63.5 ± 12.8; body mass index = 29.9 ± 6.2 kg/m^2^; 43.6% male). AAE occurred in 8.8% of primary and 6.7% of revision cases (*P* = .385), with no significant differences in major (7.6% vs. 5.6%), minor (2.4% vs. 0.6%), overall infection (3.3% vs. 1.7%), or composite surgical site infection (2.6% vs. 0.6%) subgroupings. Mean operative time (160.6 ± 60.0 vs. 165.5 ± 88.6 minutes) and total length of stay (2.3 ± 3.3 vs. 1.9 ± 3.6 days) were also all similar between groups (each *P* > .05). In the primary TEA regression, operative time (OR = 1.007) was the only independent predictor of AAE after multiplicity correction, whereas in revision TEAs, chronic steroid use (OR = 11.124) was the sole predictor (both corrected *P* = .005). A covariate-adjusted operative time cut point of ∼263 minutes (95th percentile) was identified in primary TEAs, above which AAE increased significantly (7.6% vs. 30.0%; corrected *P* = .004).

**Conclusion:**

Across this contemporary 10-year national cohort, primary and revision TEAs appeared to show comparable short-term outcomes. These findings cautiously support focused perioperative counseling and suggest that prolonged operative time becomes clinically relevant only at the upper extremes, though incrementally longitudinal, prospective studies are needed for corroboration.

Total elbow arthroplasty (TEA) has become a versatile procedure for an expansive set of indications, harboring a current incidence of approximately 1.4 per 100,000 individuals annually, that is further projected to climb in the coming decades.^18^^,^^43^^,^^45^^,^^47^ As TEA utilization has increased, revision TEAs have risen in parallel, coinciding with greater fracture and noninflammatory utilizations to supplement the more routine rheumatoid arthritis cases.^11^^,^^20^^,^^40^^,^^46^ Although TEA can yield satisfactory long-term functional and benchmark-based outcomes, there remain concerns regarding complications: a consortium that has been documented to include infection, periprosthetic fracture, and implant failure, among many others.^7^^,^^22^^,^^33^ Notably, long-term follow-up studies have demonstrated complication rates exceeding 16% and with substantial revision burden, reinforcing a paradigmatic understanding that TEA procedures are associated with considerable post-operative risk and, when compared to various other total and revision joint arthroplasties, fairly high complication rates.^6^^,^^7^^,^^22^^,^^31^^,^^45^

In the short-term post-operative period, 30-day adverse events following TEA remain clinically relevant; however, juxtapositions between primary and revision cases, as is customary in various other arthroplasty domains, are sparse in the current era.^2^^,^^25^ Much of the work from prior decades has emphasized either long-term outcomes, reimbursement patterns, or have been single-arm studies, often constrained by relatively narrow study periods, smaller sample sizes, or methodological nuances that limit generalizability, particularly regarding comparative short-term complications.^9^^,^^25^^,^^37^ Concurrently, ongoing advancements in implant designs and care protocols continually reinforce the need for additional modern, representative research that is also informative of the acute post-operative period.^3^^,^^36^ Therefore, given such considerations, the present study aims to compare short-term outcomes and associated factors between primary and revision TEAs using a large database over a more-recent 10-year retrospective window to better elucidate contemporary complication patterns and provide surgeons with updated, generalizable evidence.

## Methods

### Database features and study design

This investigation utilized the American College of Surgeons National Surgical Quality Improvement Program (ACS-NSQIP) database to identify patients undergoing primary and revision TEAs and extract associated data. NSQIP is a multicenter, prospectively collected surgical registry based in the United States that records short-term (30-day) post-operative outcomes using standardized definitions and trained clinical reviewers. Abstractable variables from this database include patient characteristics, comorbid conditions, intraoperative factors, and complication endpoints, among others. In the 2023 reporting cycle, NSQIP contained over 990,000 unique cases across nearly 700 sites and with more than 270 distinct variables.^41^ Its data integrity and validity are consistently evaluated via reliability audits, and recent reports have shown inter-rater disagreement rates of approximately 2%.^8^^,^^39^ This retrospective cohort study was conducted and reported in accordance with RECORD (REporting of studies Conducted using Observational Routinely-collected health Data), an extension of the STROBE (Strengthening The Reporting of Observational studies in Epidemiology) guidelines.^1^

### Cohort selection

Patients undergoing primary TEA between 2014 and 2023 were identified in the NSQIP database using the primary Current Procedural Terminology (CPT) code 24363 (arthroplasty, elbow; with distal humerus and proximal ulnar prosthetic replacement). Revision TEA cases were identified using primary CPT codes 24370 (revision of TEA, including allograft when performed; humeral or ulnar component) and 24371 (revision of TEA, including allograft when performed; humeral and ulnar component) during the same study interval described above, consistent with prior NSQIP-based elbow arthroplasty literature.^9^^,^^14^^,^^26^ To promote relative procedural homogeneity throughout cohort assembly, cases involving additional or concurrent procedures such as hemiarthroplasty, collateral ligament repair, fracture nonunion or malunion management, acute fracture indications, radial head excision, or any other unrelated, unspecified, or complex operations were excluded. Patients with missing or null baseline demographic data (age, sex, height, weight), or with incomplete perioperative variables, such as missing American Society of Anesthesiologists (ASA) classification, functional status, or comorbidity data, were also excluded. Additionally, to reduce confounding from potential pre-existing acute physiological instability, individuals with documented pre-operative sepsis, transfusion requirement, or ventilator dependence were removed as well. The aforementioned selection criteria were developed building upon prior NSQIP- and elbow-based outcomes investigations.^24^^,^^34^^,^^38^

### Primary outcome

The primary endpoint was the occurrence of any adverse event (AAE) within 30 days postoperatively. This composite included surgical site infection (superficial, deep, organ/space), urinary tract infection (UTI), wound dehiscence, sepsis, acute kidney injury, unplanned reintubation, prolonged ventilator support (>48 hours), deep vein thrombosis, post-operative transfusion, pulmonary embolism, pneumonia, cerebrovascular accident/stroke, myocardial infarction, cardiac arrest, return to the operating room (ROR), and death: an assortment that was assembled following previous NSQIP- and elbow-based research.^10^^,^^38^

### Adverse event subgroups

Subgroups were constructed to further characterize composite AAE and supplement between-arm comparisons. The major adverse event subgroup included death, cerebrovascular accident/stroke, myocardial infarction, cardiac arrest, acute kidney injury, pulmonary embolism, deep vein thrombosis, unplanned reintubation, prolonged ventilator support, ROR, post-operative transfusion, deep surgical site infection, organ/space surgical site infection, and sepsis. The minor adverse event subgroup was comprised of superficial surgical site infections, wound dehiscence, UTI, and pneumonia. A composite surgical site infection subgroup was tandemly created by combining superficial, deep, and organ/space surgical site infections. Lastly, an overall infection subgroup was also defined, combining all surgical site infections with UTI and sepsis. These subgroup classifications were also adapted from prior NSQIP-based, elbow-focused literature.^13^^,^^25^

### Statistical analyses

RStudio version 2026.01.0 + 392 (R Foundation for Statistical Computing, Vienna, Austria), the IBM Statistical Package for the Social Sciences (SPSS) version 31.0 (Armonk, NY: IBM Corporation), and Microsoft Excel for Microsoft 365 Apps for Enterprise version 2511 (Redmond, WA: Microsoft Corporation) were each utilized for statistical analyses. Continuous data are presented as means with standard deviations, whereas categorical/binary variables are summarized as counts (N) with percentages (%). Between-arm comparisons were performed using Pearson's chi-square testing for categorical variables with adequate cell counts, Fisher's exact testing for sparse categorical data, and independent two-tailed *t*-testing for continuous variables. Associations between covariates and the primary outcome of AAE were examined using exploratory multivariable logistic regressions, with results reported as odds ratios (ORs) and 95% confidence intervals. Initial covariate selection was guided by prior NSQIP- and elbow-based literature, emphasizing available baseline demographics, prevalent comorbidities, and surgery-related factors, with additional screenings performed to minimize redundancy, reverse causation, and multicollinearity.^25^^,^^28^^,^^38^ Notably, since regression model stability greatly depends on events per outcome, limited AAE counts precluded broad covariate inclusion. Therefore, preliminary bivariate screenings were performed, and initial covariates meeting a *P* < .250 threshold were advanced to relaxed, stepwise multivariable modeling to reduce overfitting and minimize selection bias.^4^^,^^42^ To address multiplicity arising from this stepwise approach, within-arm Benjamini-Hochberg correction was applied to findings. Regression model calibration was further assessed using the Hosmer-Lemeshow goodness-of-fit statistic. For continuous predictors showing independent associations with AAE, baseline-covariate-adjusted threshold analyses were conducted. This process involved systematically evaluating multiple candidate cut points across the variable's distribution to identify the largest statistically significant OR nearest the inflection point, thereby capturing the threshold of greatest clinical relevance. To address the resultant multiplicity also inherent to such an iterative cut point statistic, the Benjamini-Hochberg correction was accordingly applied. Throughout this study, statistical significance was assigned to any finding with a terminal *P* value less than .05.

## Results

### Cohort characteristics

A total of 583 patients underwent primary TEA (mean age = 66.4 ± 13.3 years; body mass index [BMI] = 29.9 ± 7.2 kg/m^2^; male sex = 21.3%) and 179 underwent revision TEA (age = 63.5 ± 12.8 years; BMI = 29.9 ± 6.2 kg/m^2^; male sex = 43.6%). Hypertension requiring medication was present in 56.4% of primary and 53.6% of revision TEA patients. Smoking prevalence was 12.0% vs. 17.3%, diabetes mellitus 18.4% in both groups, congestive heart failure 2.6% vs. 2.2%, chronic obstructive pulmonary disease 6.2% in both groups, disseminated cancer 1.0% vs. 0.6%, chronic steroid use 18.5% vs. 19.0%, and dialysis-dependent renal failure 0.2% vs. 0.6%, respectively. No patients in either cohort had ascites. ASA classification distribution differed between groups: 2.6% vs. 3.4% were ASA class I, followed by 38.6% vs. 40.8% ASA II, 53.0% vs. 54.8% ASA III, and 6.0% vs. 1.1% ASA IV, respectively. Among the variables described above, significant between-arm differences were only observed for age (*P* = .009), male sex (*P* < .001), and ASA classification (*P* = .031). A full summary of baseline cohort characteristics is presented in Table I.

**Table I:** Baseline characteristics and demographic details for patients undergoing primary total elbow arthroplasty (TEA) vs. revision TEA.

Cohort variable	Primary TEA mean (SD) or n (%)	Revision TEA mean (SD) or n (%)	*P* value
Patients	583	179	
Age (yr)	66.4 (13.3)	63.5 (12.8)	**.009**
Male sex	124 (21.3%)	78 (43.6%)	**<.001**
Body mass index (kg/m^2^)	29.9 (7.2)	29.9 (6.2)	.993
Race			.193
White	419 (71.9%)	125 (69.8%)	
Black or African American	22 (3.8%)	10 (5.6%)	
American Indian or Alaska Native	8 (1.4%)	5 (2.8%)	
Asian	16 (2.7%)	1 (0.6%)	
Unknown/not reported	118 (20.2%)	38 (21.3%)	
Inpatient status	344 (59.0%)	96 (53.6%)	.203
Functional status			.794
Independent	552 (94.7%)	172 (96.1%)	
Partially dependent	28 (4.8%)	7 (3.9%)	
Totally dependent	3 (0.5%)	0 (0%)	
Hypertension requiring medication	329 (56.4%)	96 (53.6%)	.509
Smoking	70 (12.0%)	31 (17.3%)	.067
Diabetes mellitus	107 (18.4%)	33 (18.4%)	.980
Congestive heart failure	15 (2.6%)	4 (2.2%)	1.000
Chronic obstructive pulmonary disease	36 (6.2%)	11 (6.2%)	.989
Ascites	0 (0%)	0 (0%)	-
Disseminated cancer	6 (1.0%)	1 (0.6%)	1.000
Chronic steroid use	108 (18.5%)	34 (19.0%)	.888
Dialysis-dependent renal failure	1 (0.2%)	1 (0.6%)	.415
ASA classification			**.031**
ASA I = no disturb	14 (2.4%)	6 (3.4%)	
ASA II = mild disturb	225 (38.6%)	73 (40.8%)	
ASA III = severe disturb	309 (53.0%)	98 (54.8%)	
ASA IV = life threat	35 (6.0%)	2 (1.1%)	

*SD*, standard deviation; *ASA*, American Society of Anesthesiologists.

### Cohort perioperative measures and complications

Patients undergoing primary TEA had a mean operative time of 160.6 ± 60.0 minutes and length of stay of 2.3 ± 3.3 days. In the revision cohort, corresponding values were 165.5 ± 88.6 minutes and 1.9 ± 3.6 days, respectively. Composite AAE occurred in 8.8% of primary TEA patients and 6.7% of revision cases. Major adverse events were observed in 7.6% vs. 5.6% and minor adverse events in 2.4% vs. 0.6%, respectively. Overall infection rates were low in both cohorts (3.3% vs. 1.7%), as were composite surgical site infections (2.6% vs. 0.6%). ROR was the most frequent individual complication in both TEA groups (4.1% vs. 2.2%). Among primary TEAs, ROR was most commonly code-categorized as post-operative infection (29.2%), followed then by musculoskeletal-related pathology (20.8%), unspecified issue (16.7%), periprosthetic fracture (12.5%), implant/mechanical failure (12.5%), wound-related pathology (4.2%), and neurological causes (4.2%). In the revision cohort, ROR drivers were evenly categorized across neurologic, post-operative infection, wound-related, and unspecified causes (each 25.0%). Notably, there were no significant differences between cohorts for any of the post-operative endpoints examined (Table II). Given the prior imbalance between age and sex across groups, a 1:1 propensity-matched analysis adjusting for baseline characteristics was also performed to further investigate potential confounding in the above-described nonsignificance (acceptable standardized mean difference [SMD] set to SMD<0.2; Supplementary Table S1).^23^ Following matching, there remained no significant differences between cohorts in any postoperative endpoints: a summary of the matched AAE and subgroup comparisons, alongside the residual balancing of ASA classification, is provided in Supplementary Table S2.

**Table II:** Short-term complication-related outcomes of primary total elbow arthroplasty (TEA) vs. revision TEA between 2014 and 2023.

Cohort variable	Primary TEA mean (SD) or n (%)	Revision TEA mean (SD) or n (%)	*P* value
Operative time (min)	160.6 (60.0)	165.5 (88.6)	.486
Total length of stay (d)	2.3 (3.3)	1.9 (3.6)	.170
Any adverse event	51 (8.8%)	12 (6.7%)	.385
Major adverse event	44 (7.6%)	10 (5.6%)	.371
Minor adverse event	14 (2.4%)	1 (0.6%)	.214
Overall infections	19 (3.3%)	3 (1.7%)	.442
Superficial surgical site infection	3 (0.5%)	0 (0%)	1.000
Deep surgical site infection	4 (0.7%)	0 (0%)	.578
Organ/space surgical site infection	7 (1.2%)	1 (0.6%)	.688
Urinary tract infection	3 (0.5%)	1 (0.6%)	1.000
Sepsis	2 (0.3%)	1 (0.6%)	.553
Composite surgical site infections	15 (2.6%)	1 (0.6%)	.137
Wound dehiscence	6 (1.0%)	0 (0%)	.345
Acute kidney injury	0 (0%)	0 (0%)	-
Pneumonia	2 (0.3%)	0 (0%)	1.000
Unplanned reintubation	0 (0%)	0 (0%)	-
Prolonged ventilator support (>48 h)	0 (0%)	0 (0%)	-
Pulmonary embolism	3 (0.5%)	0 (0%)	1.000
Cerebrovascular accident/stroke	0 (0%)	0 (0%)	-
Cardiac arrest	1 (0.2%)	0 (0%)	1.000
Myocardial infarction	2 (0.3%)	0 (0%)	1.000
Deep vein thrombosis	1 (0.2%)	1 (0.6%)	.415
Transfusion	13 (2.2%)	4 (2.2%)	1.000
Return to operating room	24 (4.1%)	4 (2.2%)	.362
Implant/mechanical failure	3 (12.5%)	0 (0%)	
Neurological	1 (4.2%)	1 (25.0%)	
Periprosthetic fracture	3 (12.5%)	0 (0%)	
Post-operative infection	7 (29.2%)	1 (25.0%)	
Wound-related	1 (4.2%)	1 (25.0%)	
Musculoskeletal-related	5 (20.8%)	0 (0%)	
Unspecified	4 (16.7%)	1 (25.0%)	
30-d mortality	1 (0.2%)	1 (0.6%)	.415

*SD*, standard deviation.

### Multivariable logistic regression analyses of AAE

Preliminary bivariate screening identified operative time, chronic obstructive pulmonary disease, smoking, age, and male sex as potential predictors of AAE in the primary TEA cohort (*P* < .250). Of these, only operative time remained as a significant independent predictor of primary TEA AAE in the multivariable model after multiplicity correction (OR = 1.007; *P* < .001; corrected *P* = .005; Table III). In the revision TEA cohort, preliminary bivariate screening identified chronic steroid use, hypertension requiring medication, diabetes mellitus, age, and BMI as potential predictors of AAE. Of this variable set, only chronic steroid use remained as a significant independent predictor (OR = 11.124; *P* = .001; corrected *P* = .005; Table IV). Based on Hosmer-Lemeshow testing, model calibration was determined to be adequate in both the primary (χ^2^ = 3.346; degrees of freedom = 8; *P* = .907) and revision (χ^2^ = 2.846; degrees of freedom = 8; *P* = .944) TEA regressions.

**Table III:** Exploratory multivariable logistic regression identifying independent predictors of AAE in the primary total elbow arthroplasty (TEA) cohort.

Primary TEA predictor	Odds ratio	95% confidence interval	*P* value	Corrected *P* value
Operative time (per min)	1.007	(1.003-1.012)	**<.001**	**.005**
Chronic obstructive pulmonary disease	1.931	(0.728-5.122)	.186	.186
Smoking	2.056	(0.885-4.774)	.094	.157
Age (per yr)	1.031	(1.005-1.057)	**.020**	.050
Male sex	1.714	(0.860-3.416)	.125	.156

**Table IV:** Exploratory multivariable logistic regression identifying independent predictors of AAE in the revision total elbow arthroplasty (TEA) cohort.

Revision TEA predictor	Odds ratio	95% confidence interval	*P* value	Corrected *P* value
Chronic steroid use	11.124	(2.615-46.860)	**.001**	**.005**
Hypertension requiring medication	5.705	(1.183-27.520)	**.030**	.075
Diabetes mellitus	0.261	(0.026-2.587)	.251	.251
Age (per yr)	1.040	(0.978-1.107)	.212	.251
Body mass index (per kg/m^2^)	0.760	(0.741-0.998)	**.048**	.080

### Threshold analyses of continuous associations with AAE

Since longer operative time was independently associated with AAE in the primary TEA cohort, a baseline-covariate-adjusted systematic threshold analysis was performed. A threshold of 262.9 minutes was identified, approximating the 95th percentile of the distribution (median = 153.0 minutes; interquartile range = 117.0-194.5). Patients undergoing primary TEAs below this time-value had a 7.6% rate of AAE compared with 30.0% above (OR = 5.792; 95% confidence interval = [2.332, 13.596]; *P* < .001; corrected *P* = .004).

## Discussion

The present study's analyses suggest that primary and revision TEAs appear to have more comparable and attenuated short-term adverse event rates (8.8% vs. 6.7%, respectively), patient profiles, infection rates, and other associated factors and outcomes than previously presumed. Operative time was the sole independent predictor of AAE in primary TEAs, with each additional operative minute associated with 0.7% increased odds of AAE, whereas chronic steroid use was the only independent predictor in revision TEAs, as patients on chronic steroids had approximately 1,102% higher odds of experiencing AAE. Threshold analysis revealed a significant operative time cut point of approximately 263 minutes (95th percentile) in primary TEAs, indicating that the more representative short-term adverse event rate in this group was closer to 7.6% for cases below such an upper-tail threshold, rather than the 8.8% rate overall. When compared with the revision TEA adverse event rate of 6.7%, this finding cautiously suggests an even narrower difference in short-term complications between groups.

In further contextualizing these findings, it is important to recognize that much of the existing literature on TEAs consists of case series, reviews, and arthroplasty registry studies, which are well-suited for survivorship and revision analyses, for example, but are less optimized for short-term perioperative benchmarking.^27^^,^^44^ Although such investigations are foundational to advancing the understanding of elbow arthroplasty, findings pertaining specifically to short-term complications and revision rates may vary substantially across the aforementioned designs. This variance can be heavily influenced by respective sample sizes, data sourcing, and methodological architecture, which will often generate obscurations that are especially meaningful when examining uncommon or rare complications.^12^^,^^31^ In contrast, NSQIP is specifically designed for short-term outcomes research, and thus provides an appropriate and robust framework for the current analysis.^8^^,^^19^

Notably, prior NSQIP-based elbow arthroplasty investigations have generated important insights into short-term TEA findings; however, analyses have historically been conducted within relatively narrower methodological and temporal frameworks. Lovy et al (2016) performed the foundational NSQIP study comparing primary and revision TEA outcomes using data from 2007-2013, arguing largely comparable 30-day adverse event rates while still identifying many modifiable and nonmodifiable risk factors such as smoking and cardiac-related comorbidity.^25^ At that time, revision procedures were identified using surrogate coding constructs due to a paucity of available revision-specific CPT codes, and the revision cohort was concurrently small (n = 53): a combination of factors that may have considerably influenced statistical results, precision, and applicability to modern elbow practices.^25^ Subsequent NSQIP investigations have examined more focused questions, though still within narrow temporal windows. For example, Sugarman et al (2021) analyzed NSQIP data from 2015-2017 and reported mainly reimbursement metrics for primary vs. revision TEA, seldom mentioning post-operative adverse events and associations.^37^ More recently, El-Najjar et al (2025) evaluated revision procedures alone using NSQIP from 2015 to 2020 and showed that revision TEA patients experience considerable early post-operative complications that are influenceable by systemic comorbidities, though comparator analyses were largely narrative due to the single-arm design.^9^ The present study's analysis of the 2014-2023 NSQIP data was conducted to supplement several of the above-mentioned studies findings and expand upon the framework established by Lovy et al (2016), delivering an assessment with the capacity for greater internal validity and statistical insights while minimizing cohort overlap. Compared to prior investigations, the present study extended the observation window and increased the size of the TEA cohorts, thereby enhancing statistical power and reducing, but not eliminating, susceptibility to biases stemming from sparse data to some degree. Importantly, the study period also began after the implementation of revision-specific CPT coding, allowing for direct identification of revision TEA cases and reducing the likelihood of misclassification bias, which may have been a more predominating relevant factor within earlier investigation iterations.

The methodological refinement conducted also helps to frame the interpretation of the presented observations relative to prior studies. Largely consistent with Lovy et al (2016), revision TEA status in the present study was not associated with a greater 30-day adverse event burden; however, in this more contemporary cohort, the differences between primary and revision procedures were further attenuated.^25^ Notably, several previously reported associations with modifiable and nonmodifiable risk factors, such as smoking, were not observed in the current analysis, and no significant difference between cohorts was observed for any specific complication, including deep infection, cautiously suggesting that earlier findings where significant differences were observed may have reflected temporal, sampling, or coding-related variability rather than persistent risk divergence.^25^ Other variations likely reflect differences in era of care, as perioperative management, implant technology, infection prevention strategies, patient counseling, and national TEA utilization patterns have all continued to evolve, and each of these factors may have played independent or compounding roles in conferring findings.^40^^,^^44^

When examining risk factors within the revision cohort, the sole-identified association appears to demonstrate biological plausibility that is consistent with prior TEA- and broader orthopedic-based literature. Chronic steroid use often reflects greater systemic burden and underlying autoimmune conditions and has been associated with increased perioperative risk when studied in surgical settings.^17^ Relatedly, immunosuppression, which may be another interpretation of this variable within NSQIP, has also been linked to short-term complications in prior TEA analyses.^26^ From a patient counseling perspective, this suggests that targeted medical optimization may be especially important in this population, particularly given the magnitude of the associated increase in adverse event risk. However, relative caution is warranted when interpreting such associations given the potential for overfitting within the dataset. In contrast to chronic steroid use in the revision cohort, operative time emerged as the sole relevant perioperative factor in primary TEAs, and longer operative durations have also been consistently associated with increased post-operative complications across both elbow and various other surgical disciplines.^38^ Within this framework, the present study's threshold-based modeling of operative time (∼263-minute cut point; 95th percentile) may be more clinically interpretable than the very modest odds ratio alone, as it further accentuates that for the vast majority of primary TEA cases, risk increases gradually and only becomes more meaningful at the upper extremes of operative duration. While it can be argued that procedure length may represent a potentially modifiable risk factor in elbow arthroplasty when setting-appropriate and amenable to optimization, the present data suggests that this factor becomes more relevant only at extreme operative durations near the inflection point in adverse event rates (7.6% below vs. 30.0% above).^5^ Furthermore, at such longer operative times outside the norm for primary TEAs, it cannot be excluded that greater case complexity and complication-prone operations themselves are not contributing to this operative time-AAE association, and thus such a finding should be interpreted with additional caution despite efforts to maintain procedural homogeneity during the cohort selection process. Accordingly, future prospective studies should investigate how surgeon experience and case volume, for example, relate to adverse events specifically in the setting of prolonged TEAs, as prior evidence suggests greater procedural familiarity may be ameliorative.^18^^,^^29^ The effects of surgical indication, precise infection microbiology, and other relevant factors should also be evaluated, when clinically ascertainable, to better refine the understanding of primary vs. revision TEA outcomes and their associations throughout the medium- and long-term.^12^^,^^15^^,^^44^

While this updated analysis positions primary and revision TEA as broadly comparable in contemporary short-term findings and associated factors, several limitations warrant consideration and must be acknowledged. Foremost, NSQIP is inherently structured around standardized 30-day outcomes and therefore does not permit evaluation of medium- and long-term complications that ultimately drive much of the patient burden associated with TEAs. And although the database employs robust abstraction and audit processes with historically low disagreement rates, participation remains voluntary, and miscoding or misclassification of operation- and complication-related variables cannot be entirely ruled out.^21^^,^^32^^,^^35^ Additionally, it is important to note that NSQIP lacks a certain degree of procedural granularity in CPT-based querying, most notably with respect to factors such as infection microbiology, implant characteristics, bone loss severity (if present), soft tissue and muscle management strategies unique to elbow arthroplasty, and various surgeon- and hospital-related metrics that may have further influenced observations. Furthermore, as is fairly common in large administrative and clinical databases, comorbidities are recorded as binary variables without accounting for severity, chronicity, or treatment modality/intensity, adding additional complexity to the possibility of residual confounding despite appropriate statistical adjustment.^25^^,^^32^ Lastly, the statistical findings reported in this study should be interpreted with consideration of both relative sample size and event-count constraints. While appropriate statistical testing was performed, sparse-event analyses inherently reduce overall precision and naturally extend to the nonsignificant findings, which should therefore not be taken as definitive evidence of equivalence between groups. Likewise, even with stepwise regression modeling, the presence of sparse-event predictors can still introduce risks of overfitting and model separation, which must also be acknowledged despite being commonly recognized limitations encountered in database-utilizing reports.^16^^,^^28^^,^^30^^,^^42^ Still, the present study persists as the largest and most temporally comprehensive modern short-term outcome comparison of primary and revision TEAs to date, helping to expand the broader discussion surrounding how these increasingly utilized procedures compare within the contexts of their historical complication burdens.

## Conclusion

Over the 10-year retrospective window of this analysis, NSQIP-based short-term post-operative outcomes between primary and revision TEAs appeared largely similar, with low rates of adverse events, attenuated between-group differences, and fewer associations than what had been observed in prior investigations. Increasing operative time was marginally associated with adverse events in the primary TEA cohort, whereas chronic steroid use showed a much stronger association in the revision TEA cohort. Interestingly, a ∼263-minute operative time cut point was isolated in primary TEAs (95th percentile), above which post-operative adverse events increased significantly, suggesting that operative time may be most clinically relevant only at the upper extremes rather than as a broadly modifiable risk factor or association. Ultimately, the expanded study window, increased sample size, and tailored design of this research, in comparison to prior inquiries, allow for a more complete juxtaposition of primary and revision TEAs, though measured caution in interpretation remains warranted due to inherent database and statistical limitations. Accordingly, prospective and complementary studies are needed to further corroborate these findings, improve surgeon understanding, and optimize elbow arthroplasty care.

## Disclaimers:

Funding: No funding was disclosed by the authors.

Conflicts of interest: The authors, their immediate families, and any research foundations with which they are affiliated have not received any financial payments or other benefits from any commercial entity related to the subject of this article.

## References

[1] Benchimol E.I., Smeeth L., Guttmann A., Harron K., Moher D., Petersen I. (2015). The REporting of studies conducted using Observational Routinely-collected health Data (RECORD) statement. PLoS Med.

[2] Boddapati V., Fu M.C., Schairer W.W., Gulotta L.V., Dines D.M., Dines J.S. (2018). Revision total shoulder arthroplasty is associated with increased thirty-day postoperative complications and wound infections relative to primary total shoulder arthroplasty. HSS J.

[3] Borton Z.M., Prasad G., Konstantopoulos G., Morgan M.L., Cresswell T., Espag M.P. (2021). Mid- to long-term survivorship of the cemented, semiconstrained Discovery total elbow arthroplasty. J Shoulder Elbow Surg.

[4] Bursac Z., Gauss C.H., Williams D.K., Hosmer D.W. (2008). Purposeful selection of variables in logistic regression. Source Code Biol Med.

[5] Cheng H., Clymer J.W., Po-Han C.B., Sadeghirad B., Ferko N.C., Cameron C.G. (2018). Prolonged operative duration is associated with complications: a systematic review and meta-analysis. J Surg Res.

[6] Chou T.A., Ma H.H., Wang J.H., Tsai S.W., Chen C.F., Wu P.K. (2020). Total elbow arthroplasty in patients with rheumatoid arthritis. Bone Joint J.

[7] Davey M.S., Hurley E.T., Gaafar M., Molony D., Mullett H., Pauzenberger L. (2021). Long-term outcomes of total elbow arthroplasty: a systematic review of studies at 10-year follow-up. J Shoulder Elbow Surg.

[8] Davis C.L., Pierce J.R., Henderson W., Spencer C.D., Tyler C., Langberg R. (2007). Assessment of the reliability of data collected for the Department of Veterans Affairs national surgical quality improvement program. J Am Coll Surg.

[9] El-Najjar D., Mehta A., Gupta P., Peterson J.R., Marigi E.M., Rogalski B. (2025). Revision total elbow arthroplasty is associated with a high rate of 30-day complications: a descriptive analysis of a national database. Shoulder Elbow.

[10] Gala R.J., Ottesen T.D., Kahan J.B., Varthi A.G., Grauer J.N. (2020). Perioperative adverse events after different fusion approaches for single-level lumbar spondylosis. N Am Spine Soc J.

[11] Gay D.M., Lyman S., Do H., Hotchkiss R.N., Marx R.G., Daluiski A. (2012). Indications and reoperation rates for total elbow arthroplasty: an analysis of trends in New York State. J Bone Joint Surg Am.

[12] Geurts E.J., Viveen J., van Riet R.P., Kodde I.F., Eygendaal D. (2019). Outcomes after revision total elbow arthroplasty: a systematic review. J Shoulder Elbow Surg.

[13] Gordon A.M., Ashraf A.M., Sheth B.K., Magruder M.L., Conway C.A., Choueka J. (2023). Anemia severity and the risks of postoperative complications and extended length of stay following primary total elbow arthroplasty. Hand (N Y).

[14] Gupta P., Quan T., Manzi J.E., Zimmer Z.R. (2022). Thirty-day morbidity and mortality following primary total elbow arthroplasty in octogenarians. Shoulder Elbow.

[15] Gutman M.J., Stone M.A., Namdari S., Abboud J.A. (2020). Treatment of elbow periprosthetic joint infection: a systematic review of clinical outcomes. J Shoulder Elbow Surg.

[16] Heinze G., Schemper M. (2002). A solution to the problem of separation in logistic regression. Stat Med.

[17] Hung Y.T., Hung W.K., Chi C.C. (2023). Effects of preoperative chronic steroid use on postoperative outcomes in orthopedic surgery: a systematic review and meta-analysis. Pharmaceuticals (Basel).

[18] Jenkins P.J., Watts A.C., Norwood T., Duckworth A.D., Rymaszewski L.A., McEachan J.E. (2013). Total elbow replacement: outcome of 1,146 arthroplasties from the Scottish Arthroplasty Project. Acta Orthop.

[19] Khuri S.F., Daley J., Henderson W., Hur K., Demakis J., Aust J.B. (1998). The Department of Veterans Affairs' NSQIP: the first national, validated, outcome-based, risk-adjusted, and peer-controlled program for the measurement and enhancement of the quality of surgical care. National VA Surgical Quality Improvement Program. Ann Surg.

[20] Klug A., Gramlich Y., Buckup J., Schweigkofler U., Hoffmann R., Schmidt-Horlohe K. (2018). Trends in total elbow arthroplasty: a nationwide analysis in Germany from 2005 to 2014. Int Orthop.

[21] Ko C.Y., Hall B.L., Hart A.J., Cohen M.E., Hoyt D.B. (2015). The American College of Surgeons National Surgical Quality Improvement Program: achieving better and safer surgery. Jt Comm J Qual Patient Saf.

[22] Krukhaug Y., Hallan G., Dybvik E., Lie S.A., Furnes O.N. (2018). A survivorship study of 838 total elbow replacements: a report from the Norwegian Arthroplasty Register 1994-2016. J Shoulder Elbow Surg.

[23] Lee B.K., Lessler J., Stuart E.A. (2010). Improving propensity score weighting using machine learning. Stat Med.

[24] Lee N.J., Shin J.I., Kothari P., Kim J.S., Leven D.M., Steinberger J. (2017). Incidence, impact, and risk factors for 30-day wound complications following elective adult spinal deformity surgery. Global Spine J.

[25] Lovy A.J., Keswani A., Dowdell J., Koehler S., Kim J., Hausman M.R. (2016). Outcomes, complications, utilization trends, and risk factors for primary and revision total elbow replacement. J Shoulder Elbow Surg.

[26] Lynch C.P., Garcia V.C., Grandizio L.C. (2023). The risk of early postoperative complications associated with preoperative immunosuppression in patients undergoing total elbow arthroplasty. J Hand Surg Am.

[27] Patil N., Cheung E.V., Mow C.S. (2009). High revision rate after total elbow arthroplasty with a linked semiconstrained device. Orthopedics.

[28] Peduzzi P., Concato J., Kemper E., Holford T.R., Feinstein A.R. (1996). A simulation study of the number of events per variable in logistic regression analysis. J Clin Epidemiol.

[29] Prkic A., Vermeulen N.P., Kooistra B.W., The B., van den Bekerom M.P.J., Eygendaal D. (2023). Is there a relationship between surgical volume and outcome for total elbow arthroplasty? A systematic review. EFORT Open Rev.

[30] Puhr R., Heinze G., Nold M., Lusa L., Geroldinger A. (2017). Firth's logistic regression with rare events: accurate effect estimates and predictions?. Stat Med.

[31] Ramirez M.A., Cheung E.V., Murthi A.M. (2017). Revision total elbow arthroplasty. J Am Acad Orthop Surg.

[32] Raval M.V., Pawlik T.M. (2018). Practical guide to surgical data sets: National Surgical Quality Improvement Program (NSQIP) and pediatric NSQIP. JAMA Surg.

[33] Satalich J., Smith M., Whitaker S., Hopper H., Setliff J., Savsani K. (2026). Indications for total elbow arthroplasty revision: a systematic review. J Shoulder Elbow Surg.

[34] Seicean A., Kumar P., Seicean S., Neuhauser D., Selman W.R., Bambakidis N.C. (2018). Impact of resident involvement in neurosurgery: an American College of Surgeons' National Surgical Quality Improvement Program database analysis of 33,977 patients. Neurospine.

[35] Shiloach M., Frencher S.K., Steeger J.E., Rowell K.S., Bartzokis K., Tomeh M.G. (2010). Toward robust information: data quality and inter-rater reliability in the American College of Surgeons National Surgical Quality Improvement Program. J Am Coll Surg.

[36] Siala M., Callamand G., Delclaux S., Bonnevialle N., Mansat P. (2021). Short-term outcomes of the Nexel total elbow arthroplasty. J Shoulder Elbow Surg.

[37] Sugarman B.S., Belay E.S., Saltzman E.B., Richard M.J., Ruch D.S., Anakwenze O.A. (2021). Trends in reimbursement for primary and revision total elbow arthroplasty. J Shoulder Elbow Surg.

[38] Talaski G.M., Yazdanpanah S., Smith M.S., Cassidy B.P., Satalich J.R., Vanderbeck J.L. (2025). Short-term complications of open reduction and internal fixation of olecranon fractures: a national database study. JSES Int.

[39] Trickey A.W., Wright J.M., Donovan J., Reines H.D., Dort J.M., Prentice H.A. (2017). Interrater reliability of Hospital readmission evaluations for surgical patients. Am J Med Qual.

[40] Triplet J.J., Kurowicki J., Momoh E., Law T.Y., Niedzielak T., Levy J.C. (2016). Trends in total elbow arthroplasty in the Medicare population: a nationwide study of records from 2005 to 2012. J Shoulder Elbow Surg.

[41] (2024). User guide for the 2023 ACS NSQIP procedure targeted participant use data file PUF. https://www.facs.org/quality-programs/data-and-registries/acs-nsqip/participant-use-data-file/.

[42] Vittinghoff E., McCulloch C.E. (2007). Relaxing the rule of ten events per variable in logistic and Cox regression. Am J Epidemiol.

[43] Viveen J., van den Bekerom M.P.J., Doornberg J.N., Hatton A., Page R., Koenraadt K.L.M. (2019). Use and outcome of 1,220 primary total elbow arthroplasties from the Australian Orthopaedic Association National Joint Arthroplasty Replacement Registry 2008-2018. Acta Orthop.

[44] Welsink C.L., Lambers K.T.A., van Deurzen D.F.P., Eygendaal D., van den Bekerom M.P.J. (2017). Total elbow arthroplasty: a systematic review. JBJS Rev.

[45] You D., King G., Dehghan N., McKee M., Morrey M., Sanchez-Sotelo J. (2026). Optimizing outcomes in total elbow arthroplasty. J Am Acad Orthop Surg.

[46] Zeltser D.W., Prentice H.A., Navarro R.A., Mirzayan R., Dillon M.T., Foroohar A. (2021). Total elbow arthroplasty: a descriptive analysis of 170 patients from a United States integrated health care system. J Hand Surg Am.

[47] Zhang D., Chen N. (2019). Total elbow arthroplasty. J Hand Surg Am.

