# Supplementary material for: Primary vs. revision total elbow arthroplasty: an updated analysis of short-term complications and associated factors
Source: J Shoulder Elb Arthroplast. 2026 May 12;10(3):100036. doi: 10.1016/j.jsea.2026.100036 (PMC13266015; doi:10.1016/j.jsea.2026.100036)
Supplement: Supplementary Table S1 [file mmc1.docx]

| **Cohort Variable** | **Before Matching** | | | **After Matching** | | |
| --- | --- | --- | --- | --- | --- | --- |
|  | **Primary TEA** | **Revision TEA** | **SMD** | **Primary TEA** | **Revision TEA** | **SMD** |
| **Patients (N)** | 583 | 179 |  | 178 | 178 |  |
| **Male Sex** | 124 (21.3%) | 78 (43.6%) | **0.507** | 78 (43.8%) | 78 (43.8%) | <0.001 |
| **Age (years)** | 66.4 (13.3) | 63.5 (12.8) | **0.223** | 65.2 (13.0) | 63.5 (12.8) | 0.126 |
| **Body Mass Index (kg/m^2^)** | 29.9 (7.2) | 29.9 (6.2) | 0.001 | 29.6 (6.9) | 29.9 (6.2) | 0.042 |
