# Supplementary material for: Primary vs. revision total elbow arthroplasty: an updated analysis of short-term complications and associated factors
Source: J Shoulder Elb Arthroplast. 2026 May 12;10(3):100036. doi: 10.1016/j.jsea.2026.100036 (PMC13266015; doi:10.1016/j.jsea.2026.100036)
Supplement: Supplementary Table S2 [file mmc2.docx]

| **Propensity-Matched Cohort Variable** | **Primary TEA Mean (SD) or N (%)** | **Revision TEA Mean (SD) or N (%)** | **p-value** |
| --- | --- | --- | --- |
| **Any Adverse Event** | 14 (7.9%) | 11 (6.2%) | 0.534 |
| **Major Adverse Event** | 13 (7.3%) | 9 (5.1%) | 0.379 |
| **Minor Adverse Event** | 2 (1.1%) | 1 (0.6%) | 1.000 |
| **Overall Infections** | 7 (3.9%) | 3 (1.7%) | 0.337 |
| *Superficial Surgical Site Infection* | 1 (0.6%) | 0 (0%) | 1.000 |
| *Deep Surgical Site Infection* | 2 (1.1%) | 0 (0%) | 0.499 |
| *Organ/Space Surgical Site Infection* | 4 (2.3%) | 1 (0.6%) | 0.372 |
| *Urinary Tract Infection* | 0 (0%) | 1 (0.6%) | 1.000 |
| *Sepsis* | 0 (0%) | 1 (0.6%) | 1.000 |
| **Composite Surgical Site Infections** | 7 (3.9%) | 1 (0.6%) | 0.067 |
| **ASA Classification** |  |  | 0.304 |
| *ASA I = No Disturb* | 6 (3.4%) | 6 (3.4%) |  |
| *ASA II = Mild Disturb* | 79 (44.4%) | 73 (41.0%) |  |
| *ASA III = Severe Disturb* | 86 (48.3%) | 97 (54.5%) |  |
| *ASA IV = Life Threat* | 7 (3.9%) | 2 (1.1%) |  |
